# Enacting a depoliticised alterity: law and traditional medicine at the World Health Organization

**DOI:** 10.1017/S1744552322000143

**Published:** 2022-04-19

**Authors:** Michael Ashworth, Emilie Cloatre

**Affiliations:** Kent Law School, University of Kent, UK

**Keywords:** medical law, science and technology studies, sociology of standards, traditional medicine, regulation, global health

## Abstract

This paper interrogates the depoliticising effects of a seemingly neutral regulatory drive at the heart of the World Health Organization (WHO)’s promotion of traditional medicine. Emerging at WHO in the late 1960s against a political backdrop of decolonisation and pan-Africanism, traditional medicine has continued to be promoted in subsequent decades, culminating in the latest global Traditional Medicine Strategy (2014 to 2023). Yet WHO’s promotion and acceptance of traditional medicine have also become increasingly conditional upon its standardisation and regulation – something that appears fundamentally at odds with traditional medicine’s heterogeneity. Drawing on insights from critical law and science and technology studies, we suggest that such a process at WHO has done more than simply disqualify the toxic and the dangerous. Rather, it has implicitly and explicitly marginalised and excluded those aspects of traditional medicine that deviate from scientific, biomedical ways of seeing, knowing and organising.

## 1 Introduction

This paper interrogates the progressively depoliticising effects of a seemingly neutral regulatory drive towards promoting standardised, ‘safe and effective’ forms of traditional medicine at the World Health Organization (WHO). In so doing, it draws from critical law and science and technology studies (STS) scholarship that sees law and science not as discrete, bounded systems of knowledge that only occasionally interact, but rather as an ‘intermingling’ (Cloatre and Pickersgill, 2020) or ‘co-production’ (Jasanoff, 2004) with its own political effects. We follow such scholarship in casting doubt on the self-proclaimed objectivity and neutrality of legal and scientific knowledge and regulatory decision-making, instead seeing it as value-laden and imbricated in relations of power. This is particularly evident when such a regime is brought to bear on an object as politically charged, profoundly heterogenous and culturally laden as traditional medicine. As the other systems of normative ordering to which traditional medicine is ordinarily subjected are disrupted, displaced or enmeshed with scientific standards and state-based legal regulation, the alterity inherent to traditional medicine is sanitised. Far from simply disqualifying the toxic and the dangerous, such a process implicitly and explicitly marginalises those aspects of traditional medicine that deviate from scientific, biomedical ways of seeing, knowing and organising. By applying a register of ‘naturalness, neutrality, facticity, objectivity, and inevitability’ (Jasanoff, 2017, p. 266) to traditional medicine, it effaces the history of (post)colonial contestation and the politics of knowledge in which traditional medicine is implicated. Although such depoliticisation may not be a deliberate strategy on the part of WHO, this illustrates the unexpected effects that turning to law can have on social practices.

WHO, established in 1948 as the UN’s specialised health agency, is a key player in global health governance. While its regulatory powers at the level of individual states are limited, it has been highly influential in shaping local policies, particularly in countries that are most dependent on global health resources. In this way, WHO can be argued to govern at a distance through the normative soft power of its resolutions, reports, programmes, standards, guidelines, declarations and meetings of various kinds (Rose and Miller, 1992). From its inception, WHO has primarily supported biomedical interventions, underpinned by activities such as disease eradication. However, against the backdrop of African decolonisation in the late 1960s and the ascendance of the Primary Health Care (PHC) movement in the 1970s, WHO began to engage with traditional medicine. As neoliberalisation and structural-adjustment programmes (SAPs) fatally undermined PHC in 1980s, WHO’s interest in traditional medicine appeared to grow. Indeed, WHO has continued to publish on the subject throughout the 1990s and 2000s, culminating in the current WHO Traditional Medicine Strategy (2014 to 2023), which is the most comprehensive organisational statement of interest in the subject to date. While the Strategy may appear as an endorsement of traditional medicine and its potential for health-care delivery, WHO’s promotion is also conditional upon traditional medicine’s standardisation and regulation. Only those forms of traditional medicine that regulatory bodies can ‘prove’ are safe, effective and of a sufficient quality are to be accepted. This represents a turn to a form of science-based and state-centred regulation that has not always been part of the discussion: some early WHO documents demonstrated an awareness of other normative orders in which traditional medicine was enmeshed and the potential limits to or inherent dangers of turning to state law. As legal pluralist scholars have long demonstrated, ‘different regulatory orders merit and foreclose different levels of agency, empower different stake-holders, reflect different institutional realities and draw on varying underlying values and principles’ (Forsyth, 2017, p. 233). Our interest therefore lies in the assumptions embedded in WHO’s turn to state-based, legal forms of regulation, how such regulation became taken for granted as the means by which traditional medicine can be utilised and what this ‘does’ to traditional medicine as a field of social practice. One particular effect is to transform the politics of the field, turning what was initially approached as a highly charged matter of culture and identity into one that can be administered through scientific standards and state bureaucracies.

This paper is structured as follows. First, we introduce the concept of traditional medicine and provide a brief historical overview of its political nature, noting its marginalisation by the colonising force of biomedicine in different contexts. We also examine the challenges that the regulation of traditional medicine raises. Second, we introduce the key ideas that lead our analysis, drawn from the sociology of standards and standardisation, and we explore its implications for socio-legal analysis. Specifically, we build on the interface between standards and politics to foreground our subsequent analysis of the depoliticising effects of regulation on debates around traditional medicine. Third, we introduce our methods and the scope of our dataset, which examines the period between 1969 – when the first resolution on traditional medicine was passed by the World Health Assembly (WHA) – and 2014 – the start of the latest Traditional Medicine Strategy. Fourth, we analyse five broad moments through which traditional medicine was progressively absorbed within a regulatory drive at WHO – and how, in the process, different facets of traditional medicine were legitimised while others were marginalised or out-right disqualified. We conclude by summarising our findings and reflecting on the limited impact WHO’s regulatory drive has had on the ground in many contexts.

## 2 Traditional medicine and its politics

Traditional medicine acts as a label that separates some healing practices from the purified modernity of biomedicine (Latour, 1993). It describes a vast and heterogenous field that elides easy summation, representing ‘disparate beliefs and practices that vary considerably from one movement or tradition to another and form no consistent … body of knowledge’ as a whole (Gevitz, 1995, p. 127). It encompasses different historically emergent, culturally based epistemological paradigms, with their own ways of knowing illness and facilitating healing (Fulder, 1998; Adams, 2003). In contrast to biomedicine, which largely focuses on biological explanations for illness, traditional medicine draws on a wider range of diagnostic and curative categories. These may range from individual physiological explanations through to the cosmological, along with an appreciation of the material and immaterial properties of plants, animals and minerals that may utilised for healing purposes (Bodeker and Burford, 2006). Rather than solely treating the body as a machine to be fixed, traditional healing may also draw on social, spiritual, ancestral and ecological factors. Although biomedicine is constituted through a scientific epistemology, it is not science that necessarily divides biomedicine from traditional forms: some types of traditional medicine actively pursue the label of science and subject their remedies to clinical trials (Jingfeng, 1987; Adams, 2002). Indeed, the neat counterposing of modern, dynamic, scientific biomedicine with historically preserved, artisanal, culturally based, holistic traditional medicine is undermined when one learns of, for example, witchdoctors in white coats in Tanzania (Nichols-Belo, 2018) and industrial Ayurveda in India (Pordié and Gaudillière, 2014) to name a few. To be sure, some contemporary changes may be related to the effects of colonialism or globalisation. However, the literature cautions against the reification of traditional medicine, highlighting that ‘no medical traditions are inherently conservative’ and unchanging (Lock and Nichter, 2003, p. 2).

Traditional medicine also holds deeply political meanings. It involves a battle of what kind of knowledge matters and, as Langwick describes, over ‘who and what has the right to exist’ (Langwick, 2011, p. 322). Its history, both in Western Europe and in colonial contexts, is one of struggle in the face of the colonising force of biomedicine. In the European context, pre-existing, open fields of healing, in which no single paradigm enjoyed hegemony, were gradually disciplined and reorganised throughout the nineteenth and twentieth centuries, with biomedicine becoming synonymous with ‘medicine’ and all other forms of healing pushed to the margins (Griggs and Van der Zee, 1997; Porter, 1999). The ascendance of biomedicine was, on the one hand, tied to genuine scientific breakthroughs, such as the emergence of antibiotics, while on the other hand, it was intimately tied up with political and legal manoeuvring, such as professionalisation of the practice, legal protection of titles and criminalisation (Saks, 2003; Cloatre and Urquiza-Haas, 2020).

Just as it had been in Western Europe, biomedicine was institutionalised in African, American and Asian colonies through scientific, political and legal processes, with local healers, practices and knowledge subject to legal prohibition and scientific de-legitimisation (Feierman, 1985; Wahlberg, 2006; Schiebinger, 2011). However, there was a fundamental difference between the spread of biomedicine in those regions and in Western Europe: while biomedicine served particular biopolitical ends in European contexts (aiming to foster and maximise the vitality of the population), biopolitical government differed in its colonial iteration as biomedicine was spread through colonial occupation (Vaughan, 1991; Arnold, 1993; Mbembe, 2019). Where biomedicine was exported to the colonies, it was primarily to serve colonial needs to facilitate conquest, rather than to necessarily improve the health of the colonised. Colonial administrators were often suspicious of traditional medicine, particularly its spiritual dimension. This was due to its (perceived) inherent irrationality and for the political dangers it posed to colonial authority, having formed part of anti-colonial rebellions in places such as Tanzania (Langwick, 2011). Anti-sorcery or anti-witchcraft ordinances were passed in many colonies, although the scope of what and who was liable to be criminalised differed somewhat depending on the jurisdiction in question (Roberts, 1935; Mutungi, 1971; Niehaus, 2001; Mesaki, 2009). As indigenous knowledge and resources were appropriated by colonialists, local plants were dislocated from their broader healing networks and molecularised through the colonial scientific laboratory (Merson, 2000; Osseo-Asare, 2014). While scientific knowledge slowly colonised healing plants, law, too, played a role. Indigenous knowledge led to patented ‘innovations’, regarded as such because of the transformation from raw plant or decoction to industrialised and marketable pill (Wahlberg, 2008).

In the post-colonial period, traditional medicine has taken on, in some cases, a particularly strong political character, featuring as part of ‘the anti-colonial struggle and pride in cultural identity’ (Tsey, 1997, p. 1065). In other cases, governments have mobilised it in pursuit of conservative or otherwise nationalistic agendas through a discursive retrieval or revival of ‘tradition’ (Lock, 1990; Wahlberg, 2006). Others still have seemingly embraced a turn to biomedicine, demarcating its role as an agent of modern societies from that of ‘ancestral’ healing practices. In the decades following independence struggles, post-colonial governments around the world grappled with the question of whether and how to deal with traditional medicine – and many specifically called upon WHO’s assistance in this regard. Post-colonial attempts by governments to utilise traditional medicine led to sometimes fractious alliances between politicians, scientific researchers, doctors and healers (Osseo-Asare, 2016). The export potential of traditional remedies in an era of globalisation has generated further political frictions: lucrative global markets for herbal products, for example, have led some, particularly Asian countries (China and India), to adapt, industrialise and commercialise their traditional remedies for export, while others, particularly African countries, have largely been shut out. This reformulation for external consumption has affected traditional medicine for internal consumption, providing a materialist prop that superficially acknowledges (for marketing purposes) or dismisses the underlying cultural worldview of the body, health and disease (Banerjee, 2002; Foster, 2016). Attempts to utilise or legitimise traditional healers have also been met with pushback from entrenched biomedical professional bureaucracies (Green, 1988) or from laws that partially or wholly criminalise the practice: either through inherited anti-witchcraft ordinances or through laws that restrict the practice of ‘medicine’ to qualified (bio)medical practitioners (Freeman and Motsei, 1992).

## 3 Regulating traditional medicine: from pluralist normative orders to state law

One consequence of the close alignment of biomedicine with law and state, then, is that legislative regulation generally draws lines around which professions can (lawfully) practise medicine and under which conditions, and which healing materials can (lawfully) be sold and utilised as medicines. Biomedical professions are usually regulated by a statutory, self-regulating medical council, which grants authorisation for practitioners who have received recognised training, with a standardised curriculum gained from an accredited institution and have passed formal qualifying examinations. This, in turn, grants them the right to use legally protected professional titles, subject to continual, adequate adherence to a professional code of conduct. Infractions result in a disciplinary process that may ultimately lead, in serious cases, to disqualification from the ability to practise and civil or criminal liability. Regulation of medicines is generally delegated to one or more agencies that are tasked with ensuring a material or technique is safe in principle, efficacious for a given purpose and that its constituent components are of a sufficient quality to guarantee safety and efficacy. In science, such safety and efficacy are proven through predetermined procedures and standards: if those are articulated in each state through national regulatory systems, they are also based on globally agreed scientific tests and procedures, of which the double-blind randomised control trial is considered the ‘gold standard’ (Kaptchuk, 2001).

Having been subject to official marginalisation and outright criminalisation in the colonial and post-colonial periods in many places, traditional medicine has generally developed outside both global scientific standards and such formal legal regulatory structures. Instead, traditional healing has historically been regulated through alternative orders, where legitimacy was derived from other kinds of social and cultural standards, mapping medical pluralism onto a form of legal pluralism (Merry, 1988). These standards are context-specific, but examples include knowledge transmission through apprenticeship, legitimacy being derived from empirical reputation and family lineage shaping claims to authority. Codes of practice can also value secrecy – a significant regulatory tool through which healers control the training process and retain control over their healing recipes and practices, but one that also distances their approach from that of scientific demands for evidence and disclosure of processes (Langwick, 2011).

For the state, such alternative legal orders create challenges, particularly in terms of visibility and control – the latter being particularly crucial in the context of public health. As post-independence or post-revolutionary states have come to take traditional medicine seriously, they have sought new means of ordering the field and tidying up what appears, from a state legal perspective, to be messy, unruly and risky (Fassin and Fassin, 1988). However, their interventions in what constitutes a complex field of sociocultural practice is rarely a politically neutral process.

Most of the literature interrogating such state regulatory intervention in traditional knowledge has come from studies of intellectual property (Coombe, 1998; Cang, 2007; Anderson, 2009; Forsyth, 2012). There is significantly less literature focusing on state-based legal regulation of traditional medicine practice. Existing literatures have nevertheless highlighted the tensions inherent to and effects of such regulation in different contexts. Some have focused on the fundamental epistemological and/or ontological incongruence between some traditional forms of healing and law. The former may be contingent upon knowledge of spirits, magic and different cosmological realms, while the latter often entails evidentiary requirements similar, if not identical, to those of biomedicine (Bannister, 2007; Cloatre, 2019). There is a parallel tendency in the realm of criminal law. Anti-witchcraft ordinances, for example, are generally not able to take seriously the existence of a spiritual realm, governed by spiritual causation, and instead target the holding of certain materials, participating in prohibited ceremonies or making accusations of sorcery (Bibeau, 1982). Unlike other normative orders, the rationality of law and of legal regulation excludes forms of knowing, seeing and healing that are dependent upon causal explanations that are not ‘provable’. Others have highlighted a central tension in the moves towards state-sanctioned, legal regulation of practitioners: those healers who hold traditional legitimacy through other normative orders may be likely to avoid seeking state recognition (Fassin and Fassin, 1988). It could be that traditional legitimacy is more important to their day-to-day work and in the eyes of their clients, while, paradoxically, it may be quacks, the inexperienced or those otherwise lacking cultural legitimacy who seek legitimisation by law and state.

One of the issues that runs through much of this scholarship is the ambivalence of the process of regulating. If state regulation is sometimes thought of as a way to increase the legitimacy of traditional medicine, its effects in practice are more divided, resulting in the de-legitimisation and exclusion of some forms of healing and some practitioners just as others are being brought into the fold of the law. State regulation of traditional healing (e.g. Ayurveda in India) can thereby perform a kind of official sanction on some, while others (e.g. Indian bone doctors) who are left ‘unregulated’ (by the state) are de-legitimised and pushed to the margins (Lambert, 2012). State regulation itself becomes one of the many components that layer and divide ‘traditional medicine’, transforming practices and boundaries as various actors seek to adjust to new spheres of legitimacy. Even if the position of biomedicine tends to remain hegemonic in relation to traditional medicine as a whole, regulation can create or reinforce hierarchies within and between traditional healing practices. Rather than regulation being superimposed over pre-existing ways of practising healing, however, one of its effects is to transform the very practices it sets out to organise. For example, moving from other normative orders to state law may require that inherited forms of authority are ‘replaced with legally based requirements that would allow patients to “read off” the credibility of practitioners and products from certificates and labels’ (Harrington, 2015, p. 187). These are requirements with which some may seek to comply, while others prefer to remain grounded in previous normative orders. Even where healers seek to comply, they may be unable to do so because of financial and administrative burdens imposed in the pursuit of achieving certification. At a more fundamental level, state regulation requires a certain level of standardisation and fixity of diverse, fluid and disparate fields. As medical traditions (e.g. acupuncture) are inherently plural, the educational and training standards required by state regulators may, for example, legitimise one particular practice style and philosophy while marginalising others (Ijaz *et al*., 2015).

Our paper builds upon and complements this body of scholarship by focusing on the global governance of traditional medicine at WHO (Ijaz and Boon, 2018). In examining WHO’s approach to traditional medicine, we take on the ‘technicalities’ of its regulatory vision (Riles, 2005). We focus on the effects of seemingly small, inconsequential historical shifts in this vision, which increasingly imposes a standardised and bureaucratised state legal apparatus that has effects well beyond disqualifying the dangerous and the toxic. In doing so, we seek to interrogate the fundamental assumption that state-based regulation of traditional healing can be a neutral process that enables the filtering of ‘safe and effective’ traditional medicine from others, as seems to be implied in the most recent strategies of WHO. At the same time, we explore how this assumption emerged, replacing other, more political debates on the place for traditional medicine in health-care practice.

## 4 Standardisation, bureaucratic regulation and depoliticisation

Our paper builds on work at the crossroad of socio-legal studies and STS, which examines the interaction between law and science as an ‘intermingling of knowledge, practices and institutions’ (Cloatre and Pickersgill, 2020, p. 82). State-based regulation of traditional medicine is such an intermingling *par excellence*, in which seemingly politically neutral legal and scientific techniques, knowledges and practices converge on a culturally and identity-laden object. If this convergence originally aims to order or ‘tidy up’ (in a neutral fashion) a seemingly messy field of practice, its effects are also potentially transformative of the field, redefining rather than rearranging it. This false neutrality of ordering is not unique to traditional medicine and its regulation, and echoes trends visible in other fields of legal and scientific regulation.

Although we are interested in the particularities (or otherwise) of legal regulation, we also acknowledge that in the field of medicine, these cannot be entirely disentangled from scientific standards. Indeed, as far as traditional medicine is concerned, it is the intermingling between different kinds of normativities that constitutes a particular claim to the political neutrality of the ordering process. With this in mind, we also build on sociological literature on standards and standardisation, bringing it into conversation with socio-legal work on legal technicalities and bureaucratic regulation.

The sociological literature on standards begins with the premise that standards often ‘promise to bring order where there was disorder; to replace the messiness of Y with the orderliness of X’ (Berg, 1998, p. 228). They are linked to the maintenance of quality and the promotion of safety (Michael, 2010). Despite this framing, standardisation is neither a neutral process nor merely ‘technical’, and the work done by standards is not reducible to the setting of objective benchmarks: the latter are defined according to knowledge processes that are themselves inevitably subject to social shaping. For one thing, standardisation has a colonising tendency and is ‘capable of indefinite extension’ (Henman and Dean, 2010, p. 80). One need only look at the explosion of evidence-based medicine in the field of biomedicine, which has restricted the exercise of discretionary, professional judgment in favour of a bureaucratic, process-driven kind of expertise, for which all potential variables can theoretically be accounted (Timmermans and Berg, 2003).

At the same time, standards have a certain ‘inertia’, meaning that dislodging them can be difficult, if not impossible (Lampland and Star, 2009, p. 14). Standards, once established, can be reformed, amended and so on, but rarely withdrawn entirely. Once applied, there is an assumption that standards are necessary and inevitable, and somehow an improvement on any alternative. This assumption often disregards the power relations and social influences that are embedded in any standard, or indeed any normative tool. Like other ordering processes, standardisation ‘valorizes some point of view and silences another’ (Bowker and Star, 2000, p. 147). Standards imply inherently that some diversity needs to be ruled out, and boundaries drawn to exclude or include particular actors or events, according to criteria set by others (Lampland and Star, 2009, p. 8). Even when such criteria appear at first sight to be neutral or universal, they rest on knowledge-making processes that, inevitably, may ‘exclude the knowledge, perspective and experiences of certain groups’ (Higgins and Larner, 2010, p. 210). By applying socially contingent knowledge to triage, exclude and other, standards and standardisation therefore have the ability to strengthen and naturalise ‘social inequalities and professional power relationships’ (Timmermans and Berg, 2003, p. 193).

As standards become enmeshed in state-based regulation, they lend a further illusion of neutrality to bureaucratic processes. State decision-making is seen as merely another layer of technical translation of objective, apolitical knowledge – something that masks the inclusions/exclusions at stake in the production of the standards themselves. This is evident in scientific fields, where the claims of both science and law as detached from politics reinforce each other, turning substantively political decisions into seemingly ‘purely’ technical ones. This process is particularly visible, as we illustrate below, in the field of traditional medicine, exemplified here by the approach of WHO. Logics of regulation migrated from biomedical practices to the more contentious and culturally sensitive field of traditional healing. Crucially, they did so without explicit engagement with the implications for practices of traditional healing. In observing these processes, we seek to emphasise several mechanisms. First, the type of standardisation that has come to permeate scientific logics was taken as the starting point to order traditional medicine, with the consequence that the drive towards standards became irreversible. Second, national governments (perhaps as a result of the state-based composition of WHO) were seen as best suited to implement such standards, which had consequences in terms of the techniques used for their implementation. Third, ‘regulation and legislation’ was progressively reduced to a particular form of technical and bureaucratic ordering of professions and materials, which sought to recreate a universal logic against which knowledge practices could be filtered. Those who were unable or unwilling to conform to particular benchmarks, procedures or bureaucratic ordering techniques were to be filtered out and excluded. Extricating traditional medicine from such logics would require imaginations that are rendered impossible by the inherent rationality of regulatory standards, while political debates have been reduced to an apparently neutral (yet illusory) triage that solely values the proven and reliable.

If the turn to state regulation is loaded with effects, shifting the ground upon which legitimacies are built, its reliance on technical standards also has the effect of depoliticising the field, or at least shifting some aspects of its ordering away from the more political discourses that once surrounded it. The forms of state-based legal regulation of traditional medicine that are normalised and encouraged are seen as a necessary improvement to the alternatives. Yet they have a largely unacknowledged ability to do more than simply disqualify the toxic and the dangerous: they also occlude the politics of knowledge and erase questions of power, history and identity in which traditional medicine is embroiled.

## 5 Data and methods

Our dataset spans 1969, the year in which the first WHA resolution on traditional medicine was introduced, up to 2014, the year in which the second WHO Traditional Medicine Strategy was launched. We sourced documentary data on traditional medicine for this time period using WHO’s Institutional Repository for Information Sharing (IRIS). We collected and analysed the records of each (annual) WHA session, including verbatim plenary speeches, summary records of committee sessions, resolutions and reports. This also included the records, reports and resolutions passed by the Executive Board, which meets twice per year before and during the WHA. In addition to the regular documentation, we collected publications on traditional medicine by WHO, including formal guidelines, standards and strategies, as well as papers in WHO’s journal and magazine. We searched the documents for references to key terms such as ‘traditional medicine’, ‘traditional healing’, ‘traditional healer’, ‘traditional practitioner’, ‘folk medicine’, ‘ethnomedicine’ and ‘traditional birth attendant’. Some documents were not electronically searchable and were examined manually. Once we constructed our dataset, we cross-referenced WHA discussions with resolutions, reports and publications, and began to build a picture of the main trends. Given the breadth and volume of data we were analysing, we limited ourselves to collecting material on WHO’s global activities rather than the outputs and activities at WHO’s regional offices. Our data analysis suggested five broad ‘moments’ in WHO’s engagement with traditional medicine and its regulation. Rather than any individual documents standing out as particular turning points, we identify successive trends in how conversations around traditional medicines in WHO started to foster assumptions about the need and inevitability of a particular kind of state-based technical regulation as a way to order the field.

## 6 Standardising and regulating traditional medicine at WHO

### 6.1 African decolonisation and PHC: the emergence of traditional medicine at WHO

That WHO should ever have become involved in promoting traditional medicine is perhaps surprising. The organisation is and always has been primarily biomedical in composition. The Secretariat and national delegations largely comprise biomedically trained professionals, while the latter represent governments where biomedicine is generally the official, state-endorsed form of healing. WHO’s early initiatives focused on promoting ‘the highest attainable standard of health’ through technical solutions and vertical interventions, such as disease eradication, and its language and logics reflected those of biomedicine (Packard, 2016).

However, by the 1960s, the political context in which WHO (and the UN more widely) operated also reflected a dramatically changed world as compared to its inception in 1948 (Cueto *et al*., 2019). Independence movements and decolonisation had swept through Asia and were sweeping through much of Africa, resulting in ballooning UN membership. Newly independent states outnumbered former colonial powers and were able to wrest control of the agenda from them. Thus, it was two African delegations – Guinea and Congo (Brazzaville) – that formally pushed the issue when they introduced a 1969 resolution on medicinal plants and pharmaceutical production in developing countries.^1^ As Tilley highlights, their resolution emerged ‘in the wake of nearly a decade of African states’ concerted and collective activities on this front’ across UN agencies and the Organisation of African Unity (Tilley, 2021, p. 141). This is not to suggest other countries were uninterested in the topic, as Member States from other regions, such as Asia – and in particular China (admitted to the UN in 1972) – also debated the issue at WHA. Rather, it explains why African countries appeared particularly engaged from the end of the 1960s through to the 1980s. The influence of African delegates on this subject was evident even through the working definition of traditional medicine that WHO arrived at during a 1977 meeting (WHO, 1978c). It was explicitly borrowed from an earlier report by the WHO African Regional Office – and encompassed a number of features that went well beyond the biomedical:

‘the sum total of all the knowledge and practices, whether explicable or not, used in diagnosis, prevention and elimination of physical, mental or social imbalance and relying exclusively on practical experience and observation handed down from generation to generation, whether verbally or in writing …. Traditional medicine might also be considered as a solid amalgamation of dynamic medical know-how and ancestral experience.’^2^

By the mid-1970s, traditional medicine had grown an increasingly prominent profile at WHO. This could be seen with its formal inclusion within Director-General Halfdan Mahler’s vision of PHC and the creation of a permanent Traditional Medicine Programme at WHO headquarters. The PHC movement, regarded as WHO’s most radical moment, sought to fundamentally reorganise health-care delivery, particularly in global southern countries (Cueto, 2004; Packard, 2016). This reorganisation included several components. It enmeshed the promotion of health within broader efforts around political, social and economic justice by calling on Member States to address the social determinants of health, including poverty, lack of education and squalor (WHO, 1975a). It promoted stricter international pharmaceutical standards and export certification schemes to end abusive practices, such as drug dumping, in which low-quality or unsafe drugs that would fail regulatory controls in richer countries were exported to poorer countries (WHO, 1975b). It called for the creation of an Essential Drugs list (a set of necessary, inexpensive pharmaceutical substances of proven quality, efficacy and safety) to counter the flooding of global southern drug markets with profitable drugs for trivial conditions, while basic, potentially life-saving medications were absent.

Most importantly for our purpose, it criticised as inadequate the colonial model of official health care that many countries inherited (urban, hospital-based biomedicine led by qualified physicians and nurses) that left significant portions of the population, particularly in rural areas, underserved (Newell and WHO, 1975). This was in stark contrast to the relatively high number of healers in the unofficial sphere of traditional medicine, who were often rurally based, and which people continued to frequent either by choice or because biomedicine was inaccessible. Taking inspiration from China’s utilisation of so-called ‘barefoot doctors’, WHO called on Member States to enlist traditional healers (including Traditional Birth Attendants, or TBAs) as part of a wider drive of utilising all available resources to meet the PHC needs of the people and to achieve the ambitious goal of Health for All By 2000. Traditional healers were seen by WHO as a valuable source of culturally acceptable ‘health manpower’ that could be added to the ‘base of the health pyramid’ (Pordié, 2010, p. 59). Healers would continue to deliver traditional medicine, but would, in addition, receive basic biomedical training. It was envisioned that this would enable them to perform limited biomedical tasks (such as dispensing anti-malarials or referring complicated cases to clinics or hospitals) and to discourage any ‘harmful’ traditional healing practices (such as not using a sterilised instrument to cut an umbilical cord). Such training did not, however, seek to penetrate and reorganise the practices and knowledge transmission of healers: that which was not actively harmful according to biomedical knowledge was left undisturbed.

One of the striking aspects of the discussion at this stage was that whether focusing on medicinal plants in 1969 or expanding the analytical scope to include traditional healers in the mid-1970s, state-based, legal regulation did not appear as the obvious solution to dealing with any potential messiness surrounding traditional medicine. Legislation was explicitly marginalised following the 1969 resolution in its accompanying 1971 Director-General’s report, which contained a short annex on traditional medicine – the first institutional consideration of the subject. Briefly discussing that traditional remedies were, in some Member States, subject to drug control legislation, Marcolino Candau, the then Director-General, observed that the contribution of such measures could not be evaluated, given that so little was known scientifically about traditional remedies (WHO, 1971, p. 7). While it was recognised in the mid-to late 1970s that regulation of healers should be considered, there was no clear, overall positive regulatory vision in which law would play a central role. Indeed, some documents highlighted that law could be an impediment to utilising healers, given that traditional healing was criminalised in many jurisdictions. A 1977 meeting on promoting and developing traditional medicine, for example, observed that law tended to encourage healing monopolies and protected the entrenched medical system – that is, biomedicine (WHO, 1978c, p. 19). Elsewhere, it encouraged healers to form ‘clubs’ or ‘societies’ to improve their practice, although law did not feature as part of the professionalising vision (WHO, 1978c, p. 39).

The most extensive consideration of legislation took place in a 1975 guideline on training and utilising TBAs, although it was ambivalent as to the positive role that law could play (Verderese *et al*., 1975, pp. 32–33). It asserted that productive legal regulation, such as light-touch registration and some form of periodic supervision by a clinic, could help improve the practice of TBAs. But it was also keenly aware of the potential danger in turning to law, which could disrupt the delicate normative cultural web in which the TBA operated, alienating her from her community. Any state-based legal regulation must be preceded by extensive education and collaboration with the communities and TBAs in question. This caution around the potentially disruptive effects of state law, as we will see in the following sections, would slowly begin to unwind over the coming years.

### 6.2 Regulating for PHC: standards for plants, law for healers

The 1978 Alma Ata Conference on Primary Health Care was arguably the zenith of the PHC movement. It issued the landmark Alma Ata Declaration (WHO, 1978a), signed by 134 national governments, which called upon its signatories to promote the radical reorganisation of health care along PHC lines, including the utilisation of traditional medicine (Rifkin, 2018). It appeared to encapsulate the optimism of the era: that Health for All By 2000 was achievable if states were prepared to be bold and to fundamentally change their conceptualisation of health-care delivery. However, 1978 was also the year in which WHO’s promotion of state-based regulation of traditional medicine began to take a more definite shape, embedding assumptions that would come to limit the possibility of alterity in healing.

In addition to the Declaration, the Alma Ata Conference issued a number of recommendations around meeting the needs of PHC. They contained a relatively underappreciated aspect: that *legislation* was a necessary tool to ‘permit communities to plan, manage and control’ PHC and ‘to allow various types of health workers to perform duties hitherto carried out exclusively by health professionals’ (WHO, 1978b, p. 76). That legislation was a central tool for PHC and, by extension, for regulating traditional healers, who were now to engage in basic biomedical tasks, was solidified the following year, when Mahler relaunched WHO’s long-standing Health Legislation Programme. Mahler’s report on the subject, published in 1979, highlighted that legislation had the potential to facilitate or derail the goal of Health for All By 2000. Importantly, he linked the issue to the legislative legacies of colonialism: many countries had ‘defective or incomplete’ health legislation and some countries retained colonial legislation that was ‘inappropriate to present conditions’ (WHO, 1979b, p. 4). WHO could assist such countries in preparing health legislation in a number of areas, including the regulation of health manpower, given that PHC had brought new categories of workers – such as traditional healers – under the purview of the state. While it gave no concrete suggestions as to which kinds of legal regulatory techniques could be applied and to what end, Mahler’s report (which was endorsed by WHA in 1980) was significant. State law was now framed in general terms as a necessary part of the toolkit for the organisation and regulation of traditional healers if the goals of PHC were to be realised by 2000.

At around the same time, the issue of how to optimise the use of medicinal plants was also receiving attention, as the 1978 Health Assembly passed WHA31.33 (Medicinal Plants) that called for a plethora of international and national standards. This included standardised botanical nomenclature; standards for demonstrating the safety and efficacy of medicinal plant products; standards for the identity, purity and strength of such products; and standardised directions for their use. WHO did not, at this stage, call for legal techniques such as registration or systems of pharmacovigilance for manufactured herbal products. Instead, the production of national and international standards of different kinds were seen as a bulwark against any potential dangers in their utilisation. On the one hand, such a move was in keeping with earlier PHC efforts by WHO around pharmaceutical standards, ensuring that poorer countries should not be subject to low-quality or dangerous pharmaceuticals or therapeutic substances. Similarly, many African governments saw plants in highly political terms as a commodity to be exploited in order to achieve post-colonial, pan-African self-reliance (Langwick, 2010). On the other hand, this was in some respects the first depoliticising move in relation to traditional medicine at WHO. The call for standards already began to foreclose the possibilities for medicinal plants by objectifying them, implicitly calling for their divorce and dislocation from their social contexts, and for privileging explicit, scientific knowledge about them, generated through a laboratory, over other kinds of tacit knowledge. Calling for such standards implied a severing of the link between healer and plant, thereby reducing the latter to little more than ‘materialist props’ (Janes, 1999, p. 1803). It also represented a clash of normative orders. The moves towards the soft law of international standards required healers’ knowledge to be made explicit, fixed and verified by scientific analysis, which could run counter to their own standards of secrecy and understandings of remedies as custom-made rather than universal. The vision promoted in the resolution was ‘a sanitized form of “natural or herbal medicine” … devoid of any “magical” elements such as spirits, divination or ancestral knowledge’ (Nichols-Belo, 2018, p. 722).

There were some cautious voices at WHA, who recognised the potential incongruence of scientific standards with traditional medicine and warned that there was a ‘danger if standardisation and scientific criteria were applied traditional systems would be lost’ (WHO, 1979a, p. 83). However, the approach expressed in the resolution was consistent with the approach taken by many national governments in their own contexts. For example, Ghana, one of the co-authors of the resolution, had recently set up the Centre for the Scientific Research into Plant Medicine. Having done so much around standards relating to drug safety and quality in the 1970s, it appeared to some at WHA that the same standards should logically apply to medicinal plants. As one member of the Executive Board expressed in January 1979 in a discussion of standardising and rigorously testing ‘indigenous drugs’: ‘[t]here can be no double standards when health is at stake’ (1979a, pp. 80–81). Although the call for technical standards and state law were part of the high politics of PHC, their effects were perhaps more paradigm-shifting than anticipated. Once the standardising and legislative regulatory logic were introduced, to plants and healers respectively, it became increasingly difficult to imagine other ways of understanding, organising and approaching these facets of traditional medicine.

### 6.3 The slow demise of PHC and continued calls for standards and law

As the 1970s gave way to the 1980s, the global political economy underwent a drastic transformation. The ‘decolonisation, democratisation and self-reliance’ of the previous decades were, in the 1980s, replaced by the growing pressure of World Bank-driven policies (Medcalf and Nunes, 2018, p. 417). SAPs gutted health-care budgets and infrastructure, particularly in the African Region. The ideology of PHC and its expression in the Alma Ata Declaration came under attack throughout the 1980s. In light of the new neoliberal paradigm, PHC was seen as too costly, unfocused and unsuited to the type of auditing that was newly favoured in global health. Mahler continued to fight for his vision of PHC and achieved some notable successes during this period, particularly around essential drugs and their regulation. From 1980 onwards, WHO convened biennially the International Conference on Drug Regulatory Authorities (ICDRA), open to all Member States, which aimed to discuss regulatory issues around pharmaceuticals and share best practices. The launch of the influential Revised Drug Strategy in 1986 saw Mahler focus on the ‘rational use’ of drugs, through promoting national drug regulations as a means of improving safety, efficacy and quality (t’Hoen, 2010). In 1987, however, amidst continuing attacks on PHC and an increasingly difficult financial environment both within and outside WHO, Mahler chose not to run for re-election as Director-General and his final term ended the following year. His replacement, Hiroshi Nakajima, took over in 1988.

The slow demise of PHC over the decade did not cause a retreat from traditional medicine. On the contrary, the deleterious effects of SAPs on national health-care systems appeared to incite and galvanise governmental interest, with new meanings becoming attached to traditional medicine and its substance and boundaries continuing to be negotiated in the process. One expression of this renewed interest was linked to the growing perception of plants as pharmacological resources. From the mid-to late 1980s, the world was in the midst of a renewed bioprospecting surge, as technological advancements in laboratory testing of plant material permitted screening ‘with a speed and precision never before possible’ (Miller, 2015, p. 52). The position of healers was slowly transformed: they were gatekeepers of plant knowledge that was not necessarily seen as valuable in its own right, but could provide a useful stepping stone for researchers seeking to access powerful plant resources. That many global southern countries were potentially sitting on ‘green goldmines’ generated further tensions between governments, communities, healers and scientists – and between global northern and global southern states (Hayden, 2003; Das, 2020). It also highlighted that plants were a finite resource in need of protection, leading to new international conservation efforts such as 1988’s Chiang Mai Declaration on Biodiversity – a collaboration between WHO, the World Wildlife Foundation (WWF) and the International Union for the Conservation of Nature (IUCN).

Against such a backdrop, then, the narrative was shifting at WHO, from the rehabilitation of culture and the high politics of PHC and its reorganisation of health care, to more practical concerns of crumbling health infrastructure, drug and personnel shortages. At the same time, the perceived necessity for national and international standards around medicinal plants and state-based regulation of healers was emphasised.

#### 6.3.1 Health legislation and traditional healers

Following the relaunch of the Health Legislation Programme, the promotion of state-based regulation of traditional medicine – particularly of healers – was proposed in different WHO documents from the early to mid-1980s. Legislative regulation had grown a sufficiently prominent profile during this period that WHO published a comparative four-part typology of regulatory regimes around the world, as part of a guide for health administrators and authorities (Bannerman *et al*., 1983). These ranged from monopolistic systems, exemplified by France, which prohibited through criminal law anyone without a medical degree from carrying out acts such as treatment or diagnosis, through to fully integrated systems, exemplified by China, which officially integrated pluralist approaches to health within its biomedical system (Stepan, 1983). Papers were published in *World Health* (WHO’s official magazine) and *World Health Forum* (its official journal) touching upon the legal status of TBAs and debating the necessity for their regulation. A consensus was emerging that monopolistic regimes were ineffective and endangered patients by driving healers underground. Yet even where there was no formal prohibition through criminal law, the legal ambiguity over the status of healers generated civil liability issues in the minds of some. What were the legal limits of their areas of competence? What constituted malpractice when it came to traditional medicine? Who would be legally liable in such a scenario – the healer, a supervising biomedical physician or nurse, or the state itself (Owens, 1983)?

Furthermore, amidst a growth in the number of healers as a consequence of soaring biomedical health-care costs, charlatanism was reportedly on the rise. Some delegates at the WHA called for the issue to be resolved expediently through the development of national policies and legislation, before traditional medicine as a whole became discredited (WHO, 1985b, p. 110). The shift from other normative orders to state law in relation to traditional medicine appeared to necessitate the ‘tidying-up’, fixing and settling of the status of the healer, in a manner akin to the biomedical professions. That healers were regulated through other culturally based normative systems began to appear less relevant as official legal logics took hold. Patients were seen as in need of protection from substandard care, practitioners from prosecution or malpractice lawsuits, and communities from charlatans – and more state law was assumed to be the solution (Owens, 1983; WHO, 1985a; 1990b).

Progress was initially slow – a situation that a 1985 WHO-convened consultation on developing national policies for traditional practitioners sought to address (WHO, 1985a). Abstracting recommendations from eight country-level case-studies drawn from Africa, Asia, the Americas and the Middle East, it called on governments to create a flexible, facilitating umbrella legislation that would unambiguously recognise the right of healers to practise (WHO, 1985a, p. 11). Yet, state regulation was still approached cautiously. Seeking to promote trust between healers and the state, the report emphasised that top-down legal regulatory tools, such as registration, may be counterproductive. Enabling legislation should not specify how to organise traditional healers or even what place they should occupy in the delivery of health care. Drawing up lists of healers was advised instead and proposed as the basis of a future registration process, once trust had been established (WHO, 1985a, pp. 9–10). The report did not suggest the sort of formal registration and training processes that would later become seemingly self-evident. Healers should instead be encouraged to form self-regulating associations, albeit supported by legislation, ‘the same as [legislation] supports councils of physicians, nurses, health auxiliaries, and other health personnel’ (WHO, 1985a, p. 12). Healers’ associations, intended to ‘establish, regulate and monitor job performance’, would ‘undoubtedly’ protect patients and the community (WHO, 1985a, p. 11). Legislation, it suggested, could lend ‘authority to the codes of ethics set by the associations’ (WHO, 1985a, p. 12). The report was not prescriptive about the composition of such associations: it would depend upon the ‘types of practitioner, as well as the social, cultural and geographical factors in each country’ (WHO, 1985a, p. 11). It suggested that there may be a need for more than one association, depending on the types of practitioners, and where there existed multiple associations, it may be necessary to organise them into an official umbrella federation, which could become an interlocutor for the government in future legal reform processes.

The report represented a development in the enrolment of traditional healing within state legislation and an incipient state bureaucratic organisation of traditional medicine. The organisation of traditional healers was slowly being reimagined in a manner akin to scientific, self-regulating biomedical professions with statutory backing. That the report envisioned such associations as forming a focal point for discussions with the government for any future changes to the law was already creating divisions and exclusions. Those able to unify, organise and professionalise would be included within the state’s future regulatory vision, while those unable or unwilling to do so would be shut out. These divisions would also not always neatly map onto other forms of social legitimacy (Fassin and Fassin, 1988). It is important to note that, like other documents from around this time (Leslie, 1983), the report showed an awareness of the limits of state law as a regulatory tool when it came to traditional medicine. For example, it highlighted that state law may conflict with certain customs: traditional practices based on ‘spirit-belief, magic, divinations, and rituals’ may belong to the customary realm, ‘for which legislation may be inappropriate’ (WHO, 1985a, p. 10). Hence, it explicitly acknowledged that state law does not take seriously the existence of a magical or spiritual realm – which is relegated to the domain of beliefs, rather than evidence (Bibeau, 1982; Bannister, 2007). In other words, it took issue with the fact that regulating traditional medicine solely through state legislation entails a de facto triaging-out of such practices, regardless of how inclusive a government intends to be.

#### 6.3.2 Renewed focus on traditional medicine and standards for regulatory authorities

As traditional healers were being absorbed into state legislation and tidied up through the promotion of tentative statutory self-regulation, calls for and activities around the standardisation of plant medicine continued apace throughout the rest of the decade. The heightened attention given to medicinal plants was coincidental with the upsurge in bioprospecting towards the end the decade. Thus, WHA reasserted its interest in traditional medicine across three resolutions between 1987 and 1989, highlighting the necessity for utilising traditional healers (which 1987’s WHA40.33 described as ‘a vast reservoir of manpower’) for PHC purposes. However, it focused more intensively on medicinal plants during this period (describing them in the same 1987 resolution as ‘an almost untapped wealth of medicinal flora’). Indeed, the 1988 resolution (WHA41.19) was solely devoted to the Chiang Mai Declaration on Biodiversity signed that year and called on Member States to realise its aims and objectives around conservation. Both the 1987 and 1989 resolutions called on Member States to promote standards: the former around quality control of drugs derived from plants by applying ‘modern techniques’ and good manufacturing processes (GMPs), the latter (WHA42.43) simply calling for ‘suitable standards’ to be applied to medicinal plants and products derived from them. The latter resolution went further, however, as it called on Member States to ‘introduce measures for the regulation and control’ of such materials.

While the resolution gave no specific indication of what such regulatory controls should look like, it reflected an intertwining of scientific standards and state regulation of medicinal plants and herbal remedies that was evident across different WHO fora during this time. This included ICDRA’s fourth (WHO, 1986) and fifth (WHO, 1991a) meetings in Tokyo and Paris, respectively, and WHO co-convened capacity-building workshops in Bangkok (WHO and DANIDA, 1986) and Harare (WHO and DANIDA, 1991). Scientific standards and state regulations became increasingly enmeshed. ICDRA’s remit had been limited to regulatory issues for pharmaceuticals, but the growth in international trade in manufactured herbal remedies resulted in calls at the 1989 meeting for WHO to publish international standards to assist national regulatory authorities in dealing with this difficult object. With more herbal products becoming manufactured, the quality and safety of medicinal plants were becoming a matter of shared global standards rather than local practices. ‘That many traditional remedies are of therapeutic value is no longer open to serious doubt,’ its report asserted, ‘but the use of manufactured products should be governed by the same standards of safety and efficacy as are required for modern pharmaceuticals’ (WHO, 1991a, p. 33). Although such standardisation went hand in hand with the expansion of global markets in herbal remedies, the assumptions on which it is built were not insignificant for traditional medicines. The incommensurability of the randomised control trial, the gold standard of adducing pharmaceutical efficacy, is well documented. Its assumption that active ingredients can be isolated and tested for measurable, clinical improvements over a relatively short period is at odds with practices that see herbal remedies as working in a synergistic fashion, over a significantly longer time period, or addressing underlying causes (emotional, spiritual and so on) rather than symptoms (Droney, 2016). WHO’s capacity-building workshops went further by explicitly marginalising traditional or indigenous knowledges when it came to safety assessment. So-called ‘folklore information’ may give an initial indication that a remedy was worthy of exploration, but it could not, ‘no matter how ancient’ the knowledge, be taken as an ‘absolute guarantee of safety in view of present-day knowledge and technology to assess safety levels’ (WHO and DANIDA, 1986, p. 7). The privileging of biomedical scientific knowledge was odds with a small number of delegations, such as Malawi, who emphasised that traditional medicine could not simply be reduced to its scientifically valid components: ‘[a]ny understanding of the totality of traditional medicine and healing methods should encompass a realisation of the fact they reflected a highly complicated value system’, the Malawian delegation stated in 1987, and ‘the portion of traditional medicine amenable to modern scientific scrutiny was only the tip of the iceberg’ (WHO, 1987, p. 158). Yet by the mid-to late 1980s, the implicit marginalisation of non-biomedical knowledge of plants enacted in 1978 had given way to an explicit hierarchy of evidence with ‘folkloric’ knowledge at the bottom.

### 6.4 The rise of global herbal medicine

The period from 1988 to 1998, under the stewardship of Director-General Nakajima, was tumultuous for WHO (Walt, 1993; Godlee, 1994; Smith, 1995; Yamey, 2002). It had been the global health leader in the 1970s, but by the 1990s, it was in danger of being eclipsed by other institutions such as the World Bank, which had initiated its own global health programmes. A mixture of donor apathy towards WHO, particularly towards its Director-General, and the toxic effects of neoliberalisation on the national budgets of global southern countries meant that the organisation continued to face a severely constrained financial environment for much of the decade (Chorev, 2013). This had a direct impact on its activities concerning traditional medicine. The Assistant Director-General asserted in 1993 that ‘in view of the low budget WHO should focus on specific priorities’ with traditional medicine, which at present meant ‘establishing guidelines and assessing research work’ (WHO, 1993, p. 109). Much of its technical work across the decade focused on medicinal plants and herbal remedies. This was partly a response to the various calls from the end of the 1970s onwards, but was also consistent with the 1980s ‘green rush’ (Osseo-Asare, 2014) that continued well into the 1990s. This had led to significant international advocacy around the conservation of biodiversity and a major pushback against biopiracy, or unauthorised access to genetic material. The 1992 UN Convention on Biological Diversity, which established a sovereign right to a state over its natural resources, was significant turning point in this regard. Against such a backdrop, it is perhaps unsurprising that there was such a focus on herbal medicine throughout the decade, given that they appeared to hold increasingly enormous potential value for community health and for the national economy.

Thus, the value of medicinal plants – both financial and health-wise – was stressed in several resolutions passed by WHA during this time, albeit subject to regulatory validation through the scientific laboratory.^3^ In 1991, WHA44.34 (Traditional Medicine and Modern Health Care) urged Member States to foster co-operation between traditional and ‘modern’ health care, particularly ‘the use of scientifically proven, safe and effective traditional remedies to reduce national drug costs’. It also called on the Director-General to provide further technical guidance in this regard and to fully ‘exploit’ the contribution of ‘scientifically proven’ traditional medicine within all WHO programmes ‘where plant-derived and other natural products’ could lead to new therapeutics. The interest in and promotion of medicinal plants at this time were not limited to the outputs of WHA. World Health published papers extolling the financial and medicinal value of plants, noting how industrial firms were turning to rainforests ‘as a source of either potential new drugs or of compounds from which less toxic or more efficacious drugs can be developed’ (Farnsworth, 1996, p. 30). Another paper discussed the growing global market for herbal medicines and noted that safety and efficacy data only existed for a small number of plants and that consequently the ‘establishment and use of regulation procedures and quality control have become major concerns in both developing and industrialized countries’ (Zhang, 1996, p. 5).

Consequently, medicinal plants were subject to a dizzying array of international technical standards throughout the decade that further transformed questions of the safety, efficacy and quality of medicinal plants into a set of bureaucratic guidelines to be followed by national regulatory authorities, manufacturers and others (WHO, 1990a; 1991b; 1998a; 1999b; 2007b; WHO *et al*., 1993). For example, WHO’s *Good Manufacturing Practices (GMP)* guidelines, the long-standing certification scheme covering pharmaceutical products intended to promote quality, were extended to cover herbal remedies. Similarly, guidelines such as 1998’s *Quality Control Methods for Medicinal Plants* provided several hundred pages of guidance on performing laboratory-based analysis of plants. Each of the technical guidelines sought to control various aspects of the life-cycle of a herbal remedy, such as storage, processing and laboratory testing, in increasingly granular detail, such as what size of instruments to use for which test. The promotion of such technical standards continued the trajectory towards a molecularised, laboratory-based approach to medicinal plants – and the marginalisation of all others – initiated at the end of the 1970s. Moreover, the blurring of the line between scientific standards and the state regulation of plants initiated in the 1980s continued during this period. For example, the 1991 guidelines on the assessment of herbal medicine – produced in response to the 1989 ICDRA request for international standards – were to serve as a model for regulators, as they contained ‘basic elements of legislation designed to assist those countries wishing to develop appropriate legislation and registration’ of herbal medicines (WHO, 1991b, p. 1).

Moreover, the technical standards were to be given statutory, regulatory backing, as WHO began promoting national regulations. While not explicitly calling for the harmonisation of regulatory standards surrounding herbal medicines, WHO published a global review of regulations in 1998, intended to facilitate regulatory information exchange and sharing of best practices. Thus, it provided an overview of over fifty countries’ regulatory regimes, noting, for example, the different regulatory standards triggered by the classification of a herbal remedy as a food supplement or a medicine (WHO, 1998b). It was critical of the weak or non-existent regulatory regimes and suggested that there must, at the very least, be some kind of special licencing regime, which would enable the management of risks to safety, including through post-market surveillance (WHO, 1998b, p. 1). This would permit those herbal remedies making therapeutic claims for minor conditions to be subject to some form of control, without imposing the most stringent, pharmaceutical-style regulatory burdens on manufacturers of herbal remedies.

### 6.5 Global, well-regulated, depoliticised traditional medicine

Beginning with Gro Brundtland’s single term as Director-General (1998–2003), WHO’s fortunes were revived (Cueto *et al*., 2019). Brundtland oversaw the implementation of wide-ranging reforms that not only re-established WHO as a credible player in global health, but successfully redeployed the logic of neoliberalism to argue for increased spending on health care by donor countries and organisations, as a matter of macroeconomic good sense (Chorev, 2013). This was, however, a highly targeted form of spending, underpinned by the notion of cost-effectiveness. Money was poured into health interventions that could be measured through quantitative data and for which significant statistical improvements across particular metrics could be demonstrated. One casualty of this renewed focus on cost-effectiveness was the TBA programme – one of the primary figureheads of the 1970s PHC movement and its overlap with traditional medicine. In a 2005 WHO *World Health Report*, dedicated to the topic of maternal health, WHO declared its own TBA programme a failure (WHO, 2005). It highlighted several fundamental issues with the programme, including a failure to understand the ‘immense cultural gap’ between biomedical care and the activities of the TBA (WHO, 2005, p. 70). Focusing on the negligible impact that the TBA programme had had on maternal and infant mortality rates in different contexts, the report concluded that the money ‘would have been better used to train professional midwives’ (WHO, 2005, p. 70).

However, this negative assessment of the TBA programme specifically did not mean that WHO jettisoned its interest in traditional medicine or even in traditional healers. On the contrary, WHO appeared more invested in the promotion of traditional medicine than ever before. The scattered programmes, resolutions, reports and guidelines were drawn together into global strategies from 2000 onwards (WHO, 2000; 2004d; 2009). Traditional medicine formed the subject of the WHO Congress on Traditional Medicine – a major international conference held in 2008 in Beijing. Not only was WHO invested in promoting traditional medicine, but it appeared to understand its heterogeneity, as evinced in its first Traditional Medicine Strategy (2002 to 2005), which provided a definition similar in scope to WHO’s working definition in the 1970s: ‘diverse health practices, approaches, knowledge and beliefs incorporating plant, animal and/or mineral based medicines, spiritual therapies, manual techniques and exercises applied singularly or in combination to maintain well-being, as well as to treat, diagnose or prevent illness.’

It observed that traditional medicine may be:

‘codified, regulated, taught openly and practised widely and systematically, and benefit from thousands of years of experience. Conversely, it may be highly secretive, mystical and extremely localized, with knowledge of its practices passed on orally. It may be based on salient physical symptoms or perceived supernatural forces.’ (WHO, 2002, p. 7)

However, WHO’s promotion of traditional medicine at this stage was entirely conditional upon its state-based regulation and standardisation, seen as essential to guarantee safety, quality and efficacy. The consequences this might have for accommodating some key characteristics of traditional healing – its spiritual dimensions, secrecy, mysticism and the oral transmission of knowledge – were notably absent from discussions of these new legal expectations. The promotion of traditional medicine by WHO, despite its expansive definition, therefore created an implicit blindness to its own exclusionary effects that would remain in the years to come.

Moreover, the regulatory and standardising drive appeared to reach even greater facets of the practice of traditional medicine by this point. In the realm of medicinal plants, standards begot more standards that extended inwards, examining new molecular issues such as contaminants and residues in herbal medicines (WHO, 2007a), and outwards, through attempts to control the agricultural practices through which plants were produced (WHO, 2004b). Calls were made for the establishment of pharmacovigilance systems to monitor the safety of herbal remedies on the market (WHO, 2004c). Guidelines were produced that targeted the consumer of traditional medicine, seeking to promote safety by ensuring that they were ‘empowered’ to make the ‘right’ choice when utilising traditional medicine (WHO, 2004a). In the realm of healers, state law was called upon to do more than unambiguously recognise their right to practise or provide statutory backing to self-regulation, as in the 1980s. State regulation and standards began to colonise the realm of training, qualification and accreditation of traditional healing, as a means of guaranteeing the safety, efficacy and quality of practitioners and practices. Training had previously been a concern during the era of PHC, but only insofar as healers were to deliver basic biomedical care, which meant inculcating biomedical knowledge and, in the process, eliminating harmful (from a biomedical perspective) traditional practices. Traditional practices as such, and associated knowledge transmission, were largely left undisturbed. The regulatory and standardising drive had grown by the 2000s and now sought to penetrate and ‘tidy up’ the knowledge transmission of traditional practices and the healing practices themselves.

In 2010, WHO published international benchmarks for training in seven different traditional medicine disciplines: traditional Chinese medicine, Ayurveda, Nuad Thai, Tuina, Unani, osteopathy and naturopathy (WHO, 2010e; 2010a; 2010c; 2010g; 2010f; 2010d; 2010b). This was in addition to a single set of guidelines already published on acupuncture in 1999 (WHO, 1999a). The benchmarks were described as comprising what ‘the community of practitioners in each of these disciplines considers to be reasonable practice in training professionals’ and were directed towards ensuring safety, efficacy and quality (WHO, 2010e, p. vii). This was not in service of some kind of self-regulatory professionalisation, however, but was intended to help authorities establish ‘adequate laws, rules, and licensing practices’ (WHO, 2010e, p. vii). It was important, then, that ‘policy-makers’ were ‘able to standardize the training of practitioners’ as a means of ‘protecting both the providers and the consumers’ (WHO, 2010e, p. viii). The training regime envisioned in the benchmarks was highly formalised, with completed secondary education required as a bare minimum. The training requirements varied between disciplines, but generally consisted of roughly 1,000 hours of clinical supervision, in addition to hundreds of hours of study across various modules. These were internal to the discipline, but also included training in subjects such as human anatomy, pharmacology and physiology, to name a few. Moreover, the training regime was intended to be full-time for two to three years – in other words, akin to university degrees.

While this is ostensibly an attempt to enable governments to include safe, efficacious and quality traditional medicine, it entails fundamentally problematic assumptions and generates myriad exclusions. For one thing, such benchmarking appears to legitimise therapies that can be professionalised, taught and organised like biomedicine – and, conversely, marginalises all others, sometimes against the grain of local communities’ own registers of legitimacy. The preface to the benchmarks even observes that WHO chose to focus on therapies for which countries had already ‘established formal education or national requirements for licensure or qualified practice’ in their respective countries of origin; that is, they were already formally institutionalised at the national level in different contexts (WHO, 2010e, p. x). The benchmarks were a kind of scaling-up to the international level in globally popular forms of traditional medicine and a means of assisting national authorities in properly regulating such practices. This may, in principle at least, work for practices that can be (re)organised around a biomedical model of training, accreditation and licensure, but many forms of traditional medicine elide such tidying-up. Similarly, the assumption that practices can be represented by a neat community that would be willing to act as an interlocutor to the state ignores some of the tensions that animate the relationship between healers and state representative in numerous countries. Where state initiatives seek to produce the form of regulation through professionalisation suggested by WHO, those who come forward to represent ‘traditional healing’ may bear little resemblance with those entrusted by local populations or communities (Fassin and Fassin, 1988).

As evinced from the 2014 Traditional Medicine Strategy, the regulatory, standardising drive around practitioners and practices had set its sights well beyond the training of the initial seven disciplines it targeted. It calls on Member States to develop a plethora of standards that, as Wahlberg puts it, amounts to making traditional medicine ‘auditable’ (Wahlberg, 2015). These include developing indicators for monitoring job performance; ‘practice guidelines’ to ensure safety, efficacy and quality; and standards and regulations covering education, training, accreditation and reimbursement for different kinds of traditional medicine. Not only was WHO advocating the organisation of training of certain disciplines into biomedical-style higher education courses, but it was now promoting practice guidelines – a core component of the evidence-based medicine revolution in biomedicine that has been subject to intense criticism (Timmermans and Mauck, 2005). Moreover, regulations themselves are envisioned as amenable to standardisation and benchmarking: the Strategy is effusive in the power of benchmarks in this regard, as they can be used to evaluate the effectiveness of regulatory frameworks through ‘national audits and reviews as well as by developing and sharing appropriate models at the international level’. Unlike in the 1970s and 1980s, when WHO documents demonstrated an awareness of the limits of state law and when delegates openly questioned whether scientific standardisation would, in fact, destroy traditional medicine, state law and standards are in the 2014 Strategy an unqualified good.

## 7 Conclusion

As the drive proceeded towards standardisation and state-based, legislative regulation of plant medicine and traditional healers, some questions and framings disappeared from WHO debates. If in the 1960s the stakes of traditional medicine were also about identity and alterity, and about proposing futures in which different types of knowledge and different types of traditions mattered, such political debates became muted. If traditional medicine has acquired more visibility in WHO discourses, with its own global Strategy by the turn of the millennium, the conditions attached to its promotion mean that what is being promoted is a reshaped and tidied-up version – one amenable to the standards and processes of biomedicine. What is perhaps most remarkable is that it is difficult to pinpoint a specific moment at which traditional medicine moved from being about high-stakes politics to being a more tamed object of global and national policy. Instead, the increasing turn to standards and a particular type of bureaucratic state regulation seems to have progressively depoliticised the field, as the knowledge politics at the core of medical pluralism disappeared in favour of new technical standards and procedures.

The early caution around the clash of normative orders in which traditional medicine was enmeshed – and the recognition of the limits of state law – has been slowly sidelined in favour of increasingly self-confident promotion of standards and state regulation. This may be because what was imagined as state regulation also shifted over the decades, moving from discussions over criminalisation and prohibition to technical and bureaucratic forms of regulation. Yet, one consequence of such technical regulation is to make political debates disappear through mundane and seemingly neutral procedural requests. This is evident in the history of WHO’s approach to traditional medicine. A technical ‘tidying-up’ was initially directed towards medicinal plants, as the dreams of post-colonial self-reliance led to an implicit and then an explicit privileging of scientific over other kinds of knowledge. The immaterial or spiritual dimensions of medicinal plants or their implication within broader healing networks were closed off as pharmaceutical-style regulatory standards were promoted. Similarly, traditional healers became subject to standards and state regulations, rooted in bureaucratic and auditable techniques. Just as the standardisation and registration of herbal remedies have promoted a vision of medicinal plants that looks remarkably like biomedicine, so too have bureaucratic systems of registration, training and licensure given rise to a biomedical-style reorganisation of traditional healers.

The organisation of healing plants and of healers has been enacted with the aim of promoting safety, efficacy and quality. While these remain laudable goals, the limits of universal standards and state regulation as tools to achieve them deserve at least some attention. These include the sociopolitical implications of the exclusion or marginalisation of perspectives, knowledges and experiences in traditional medicine that are not provable according to biomedicine or legible according to the state. Tacit, secretive or inexplicable forms of knowledge, along with oral, familial and spiritual types of knowledge transmission, are pushed to the margins in favour of open, explicit, laboratory-generated knowledge and highly formal, bureaucratic, university-style systems of knowledge transmission. Such a process has not only marginalised some practices over others, but has potentially created or reinforced hierarchies between different styles or philosophies of what is ostensibly the same type of practice. In other words, it has progressively foreclosed and sanitised the heterogeneity, the different ways of knowing and the political challenge to the establishment represented by traditional medicine. What is left at WHO are forms of traditional medicine that, as Banerjee puts it, wink at alterity, but are included because they can be organised and validated as though they were biomedicine (Banerjee, 2004). But even if we limit our focus to undoubtedly important issues of safety in health-care delivery, the exclusionary effects of regulation require consideration: where regulation and social legitimacy are fundamentally mismatched, those excluded by regulation may be driven towards invisibility, on the edge of the law, rather than conforming or disappearing.

If the high political stakes of traditional medicine seem to have been replaced by a reliance on bureaucratic ordering, this is not the case at local levels – particularly across Africa. There, the fractious alliances between politicians, healers, physicians and scientists established in the aftermath of colonialism continue to hold, transform or collapse. The regulatory vision promoted by WHO fails to translate into the straightforward practices that it assumed possible, eliding what the delegate from Malawi recognised in 1987: that what can be subjected to scientific analysis is but a small part of a very complex cultural system of values. In other words, engaging seriously and effectively with traditional medicine requires more than simply replicating the regulatory system of biomedicine – a system that is evidently ill-equipped to deal with an object as heterogenous as traditional healing.

Finally, this paper presents a specific example of regulating an object historically designated as ‘non-science’ and governed through alternative normative orders (Brosnan *et al*., 2018, p. 8) in order to build upon and extend more general insights regarding the intermingling of law and science (Cole and Bertenthal, 2017; Cloatre and Pickersgill, 2020). While much of the critical socio-legal and STS literature has attended to the question of how law and regulation respond to the question of technological innovation (Lynch and McNally, 2003; Jasanoff, 2005), our focus here has been on the regulation of what is often regarded as innovation’s antonym: traditional medicine practice. In this way, our project diverged somewhat from those who have examined ‘traditional knowledge’ through the lens of the contemporary intellectual property regime, focusing on the politics of patenting ‘innovations’ drawn from non-Western communities (Hayden, 2003; Osseo-Asare, 2014; Foster, 2016). Building upon those who have interrogated the politics and power relationships embedded within scientific and legal knowledge, practice and institutions (Jasanoff, 1994; Adams, 2002; M’Charek, 2008), our primary contribution has been to highlight law’s limited ability to embrace ontological alterity. One of the remarkable effects of a turn to state regulation, in our example, has been to seemingly depoliticise material practices thoroughly enmeshed within the high politics of colonial and post-colonial culture, history and identity. Where science and law, particularly in the form of bureaucratic regulation, strive to be seen as detached from politics, the ordering they co-produce crowds out alternative epistemologies and ontologies, turning what may be highly culturally sensitive and profoundly political discussions into technical ones. Thus, even where genuine attempts are made to include and to work towards alternative futures, such projects may be compromised from the outset where legal and scientific knowledge alone are determinative of the boundaries of the legitimate.

## Footnotes

1 WHA22.56 (establishing pharmaceutical production in developing countries), May 1969. The wording was broadened to ‘traditional medicine’ during the committee debate.

2 Paragraphing has been altered from the original.

3 Medicinal plants were also briefly mentioned in resolutions marking the International Decade of the World’s Indigenous Peoples passed during the 1994 (WHA47.27) and 1998 (WHA51.24) Health Assemblies.
